# Pulmonary Fibrosis Related to Amiodarone—Is It a Standard Pathophysiological Pattern? A Case-Based Literature Review

**DOI:** 10.3390/diagnostics12123217

**Published:** 2022-12-19

**Authors:** Corina Eugenia Budin, Iuliu Gabriel Cocuz, Adrian Horațiu Sabău, Raluca Niculescu, Ingrid Renata Ianosi, Vladimir Ioan, Ovidiu Simion Cotoi

**Affiliations:** 1 Pathophysiology Department, George Emil Palade University of Medicine, Pharmacy, Science and Technology of Târgu Mures, 540139 Targu Mures, Romania; 2 Pneumology Department, Mures Clinical County Hospital, 540142 Targu Mures, Romania; 3 Pathology Department, Mures Clinical County Hospital, 540142 Targu Mures, Romania; 4 Faculty of Medicine, George Emil Palade University of Medicine, Pharmacy, Science and Technology of Târgu Mures, 540139 Targu Mures, Romania

**Keywords:** amiodarone, pulmonary toxicity, interstitial lung disease, diffuse parenchymal lung disease, radiology

## Abstract

Amiodarone hydrochloride is an antiarrhythmic drug, with proven efficacy in prevention and treatment of numerous arrhythmias, atrial fibrillation especially, or ventricular arrhythmias, with a long half-life (55–60 days). The increased risk of developing amiodarone-induced pulmonary fibrosis is directly related to the dose and the duration of the intake. Amiodarone-induced pulmonary toxicity is conditioned by dose, patient’s age, and pre-existent pulmonary pathologies. The pattern for drug-induced lung injury may vary in many forms, but the amiodarone can cause polymorphous injuries such as diffuse alveolar damage, chronical interstitial pneumonia, organizing pneumonia, pulmonary hemorrhage, lung nodules or pleural disease. The pathological mechanism of pulmonary injury induced by amiodarone consists of the accumulation of phospholipid complexes in histocytes and type II pneumocytes. Differential diagnosis of pulmonary fibrosis induced by amiodarone is made mainly with idiopathic pulmonary fibrosis, left ventricular failure or infectious disease. Before starting treatment with amiodarone, patients should be informed of potential adverse effects and any new respiratory symptoms should promptly be reported to their family physician or attending physician. The assessment carried out at the initiation of amiodarone treatment should include at least chest X-ray and respiratory function tests and extrapulmonary evaluation.

## 1. Introduction

### 1.1. Clinical Pharmacology

Amiodarone hydrochloride is an antiarrhythmic drug with proven efficacy in the prevention and treatment of numerous arrhythmias, atrial fibrillation especially, or ventricular arrhythmias [1], with a long half-life (55–60 days) [1,2]. Although it is classified as a class III antiarrhythmic, it has been shown that it can affect all phases of the action potential [3]. Amiodarone is metabolized to desethylamiodarone (DEA) by cytochrome P450 in the liver [4]. After the hepatic metabolism and biliary excretion, a small amount of amiodarone and DEA is found in the urine [1,4].

After the first dose, amiodarone reaches its plasmatic peak levels in 3 to 7 h. The onset of action can take from a few days to a few weeks. Biodisponibility can be influenced by age, liver pathology and interactions with other drugs or substances that can inhibit or stimulate cytochrome P 450 [5,6].

Because of the lipophilic structure, both amiodarone and its metabolites accumulate in high quantities in tissues and also interact with the phospholipid’s metabolism [1,7]. These tissues are represented by adipose tissue and well-perfused organs: liver, lung or skin tissue. The most frequent affected organs by the accumulation of amiodarone and, respectively, the high risk of developing injuries induced by amiodarone are the eyes (cornea deposits, photophobia), the thyroid gland (hypo/hyperthyroidism), the liver (drug-induced hepatitis, dyspeptic syndromes), the skin (photosensitivity) and the nervous system (peripheral neuropathy) [1,8].

Although the lungs are rarely affected (approximately 4–6% of all complications) [9], pulmonary injury has the most clinically significant impact, which can lead to the patient’s demise [8,10]. Amiodarone-induced pulmonary toxicity is conditioned by dose, patient’s age and pre-existent pulmonary pathologies [1]. Those effects reach a plateau at a cumulative dose bigger than 150 g. Patient’s comorbidities, oxigenotherapy, invasive procedures or surgical interventions can trigger the pulmonary symptoms induced by amiodarone toxicity [1,4].

Amiodarone therapy also interferes with other drug classes, such as warfarin, simvastatin, atorvastatin as well as antiretroviral medication used in patients with HIV [3,11]. Taking into account these considerations and the frequent use of amiodarone in medical practice, physicians must know the indications, contraindications, dosage, adverse effects and drug interactions of amiodarone treatment [3,11,12].

Usual doses between 200–600 mg/day have minimal hemodynamic adverse effects. They cause a negative inotropic effect related to the administered dose by reducing systemic vascular resistance [5]. It has no effect on the ejection fraction of the left ventricle, and arterial hypotension rarely occurs during oral treatment with amiodarone [6].

### 1.2. Pathology

Pulmonary fibrosis is characterized by pulmonary destruction and remodeling due to the accumulation of collagen and extracellular matrix at the tissue level [13]. They cause an irreversible decrease in lung capacity, impairment of gas exchange and hypoxemia [4,13]. Systemic or inhaled toxic agents such as bleomycin, nitrofurantoin, amiodarone or ionizing radiations can be involved as etiological factors [13,14].

Evidence from the scientific literature has highlighted the fact that cells of the innate immune system as well as the adaptive one and the mediators that these cells release cause the appearance of interstitial changes [15]. Macrophages are phagocytic cells belonging to the innate immune system [13]. Present in all tissues of the body, most commonly in the lung and liver, macrophages function as immune sentinels, with the aim of defending the body against pathogens and injuries [16]. Resident macrophages are distinct from bone marrow-derived inflammatory macrophages that accumulate in tissues in response to injury or infection [13,14]. Inflammatory macrophages are mainly involved in the development of pulmonary fibrosis [17]. Macrophages have been classified into M1-pro-inflammatory/cytotoxic and M2-anti-inflammatory/reparative [18], which develop in response to signals present in the tissue microenvironment [19].

The pattern for drug-induced lung injury may vary in many forms, but amiodarone can cause polymorphous injuries such as diffuse alveolar damage (DAD), chronical interstitial pneumonia (CIP), organizing pneumonia, pulmonary hemorrhage, lung nodules or pleural disease [20,21].

The pathological mechanism of pulmonary injury induced by amiodarone consists of the accumulation of phospholipid complexes, which contain amiodarone, in histocytes and type II pneumocytes [22]. The accumulation of phospholipids is determined by the amiodarone’s suppression of phospholipases [1,22]. The occurrence of amiodarone-induced pulmonary lesions is correlated with cumulative doses and pre-existent respiratory diseases. The majority of lesions appear at doses greater than 400 mg/day, but there are documented cases in which these can occur at doses of 200 mg/day or even less [23]. Pulmonary lesions appear mostly after 2 months of administration [20]. However, a correlation between the administered dose of amiodarone and the severity of the lesions could not be found [24]. The presence of type II pneumocytes and macrophages with vacuolated cytoplasm indicates a history of amiodarone administration, but it does not support the amiodarone-induced acute pulmonary lesion diagnosis [8]. In the bronchoalveolar lavage fluid of patients with amiodarone-induced pulmonary injury we can observe cytotoxic T cells. Amiodarone can determine the production of oxygen free radicals, which lead to cellular lysis. [4].

Characteristically, the histopathological diagnostic of amiodarone-induced lung injury is established on tissue samples, in the routine staining of hematoxylin–eosin. Microscopically, upon lung tissue analysis of the pulmonary parenchyma we can highlight diffuse interstitial pneumonitis [21]. It is described by a type II pneumocytes hyperplasia, inflammatory infiltrate within the alveolar septa and variable degrees of pulmonary fibrosis [4]. Vacuolated cytoplasm can be hilighted in alveolar pneumocytes, bronchial epithelium and endothelial cells [4,24]. The accumulation of foamy alveolar macrophages is specific to APT (amiodarone-induced pulmonary toxicity). Electron microscopy examination displays membrane-bound lamellar bodies and lipid particles such as surfactant due to accumulation of drug accumulation into the lungs [1,4,23]. Less frequent pathological manifestations are patchy bronchiolitis obliterans, organizing pneumonia or, in severe cases, diffuse alveolar injury with formation of hyaline membranes or alveolar hemorrhage induced by amiodarone [4]. For establishing a histopathological diagnostic of amiodarone-induced lung toxicity, the correlation between the microscopic aspect and the clinical data must be corelated.

### 1.3. Clinical Presentation

From a clinical point of view, the patients with amiodarone-induced pulmonary fibrosis can present different degrees of progressive dyspnea, unproductive cough, fever or, rarely, pleural pain. On auscultation, bilaterally basal “Velcro-like” inspiratory crackles are found [8]. Acute or over-acute forms are presented as acute respiratory failure, with possible onset of acute respiratory distress syndrome [24,25]. Among cases with diffuse alveolar damage, the phenomena of respiratory failure dominate the clinical picture. In cases of bronchiolitis obliterans-organizing pneumonia (BOOP), symptoms may mimic bacterial pneumonia [26,27].

### 1.4. Diagnostic

From a functional point of view, a moderately restrictive type of pattern is frequently highlighted, with a decrease in forced vital capacity (FVC) and a moderate decrease in the diffusing capacity for carbon monoxide (DLCO) in approximately 45% of patients [5,10]. A nonspecific inflammatory syndrome can also be highlighted, characterized by a mild leukocytosis, increased erythrocyte sedimentation rate and increased C-reactive protein (CRP) value, but these are nonspecific and are associated with interstitial inflammation [7,13].

Bronchoalveolar lavage (BAL) is essential to confirming the diagnosis of interstitial damage induced by amiodarone. The decrease in the number of macrophages in BAL is characteristic of amiodarone-induced alveolitis. It is a sensitive method, but not very specific. Moreover, the presence of foamy macrophages suggests amiodarone-related alveolitis but without complications.

### 1.5. Imagistic Features

Interstitial lung damage has been described as a severe adverse effect associated with some drug therapies, such as bleomycin, amiodarone or methotrexate. The mechanism by which amiodarone produces this type of lung damage is through the accumulation of phospholipids at the level of alveolar macrophages [28]. Pulmonary involvement is diverse [29]. It can be like the ground glass opacities type, with peripheral localization. Interstitial, alveolar or mixed infiltrates located bilaterally or high attenuation areas in infiltrates, lung nodules or “masses”, may be single or multiple, often peripheral in location. Dense bilateral basal reticular opacities and traction bronchiectasis suggests pulmonary fibrosis [1,4]. Upon CT examination, a severity score can be calculated, depending on the number of affected regions (right and left; upper, middle and lower; and central and peripheral). This severity score correlates with symptomatology and pulmonary functional impairment [30,31].

If amiodarone-induced lung damage is BOOP type, bilateral consolidation areas are present, located subpleural and peribronchovascularly, with patchy areas of ground glass [8,32]. The location and intensity of these lesions may vary over time, either with treatment or spontaneously [8]. In non-specific interstitial pneumonia (NSIP) pulmonary involvement, HRCT examination reveals areas of ground glass, reticular opacities and linear fibrosis. Associated, subpleural areas of honeycombing and traction bronchiectasis are present [33]. A chest X-ray reveals an interstitial lesion. On HRCT (high resolution CT) interstitial damage, reticular or reticulonodular opacities and traction bronchiectasis are present [1,25]. Honeycombing is less common than in idiopathic pulmonary fibrosis [10]. The most dramatic manifestation of amiodarone-induced alveolitis is rapidly progressive diffuse pneumonitis with acute respiratory failure and ARDS-like changes [24,25].

### 1.6. Differential Diagnosis

The main pathologies with which the differential diagnosis (Table 1) of amiodarone-induced interstitial lung damage is made are idiopathic pulmonary fibrosis, hypersensitivity pneumonitis, infectious pathologies or heart failure [33,34]. Comorbidities such as diabetes or stroke are common in these patients, which increases the risk of respiratory infections [8,35].

The differential diagnosis between bacterial pneumonia and BOOP is difficult to perform because the clinical and radiological manifestations are superimposed [8,11]. The sudden onset, purulent sputum, unilateral localization advocates the diagnosis of bacterial pneumonia. Procalcitonin is a useful marker for differential diagnosis, being elevated in bacterial pneumonia and unchanged in BOOP [8].

Early stages of interstitial damage are difficult to differentiate from cardiac stasis because patients indicated for amiodarone treatment have chronic cardiac pathologies [8,36].

Heart failure and pulmonary thromboembolism are pathologies that must be considered in patients with interstitial involvement [8]. Pulmonary thromboembolism superimposed on amiodarone-induced interstitial lung damage is difficult to diagnose and contributes to the poor prognosis of these patients [8]. Repetitive pulmonary thromboembolism in the small branches of the pulmonary artery may not have a characteristic clinical expression, but may be manifested only by mild hemoptysis, progressive dyspnea in the context of the establishment of pulmonary hypertension [34]. At the same time, subpleural consolidations due to pulmonary infarcts are difficult to differentiate from the imaging changes of BOOP [25].

Acute cardiogenic pulmonary oedema is a possible complication in patients with decompensated heart failure or pulmonary stenosis. The imaging appearance is confirmed by the appearance of Kerley lines and peribronchial oedema [29]. Subpleural patchy areas also occur in pulmonary oedema. The clinical expression is what differentiates the diagnosis at patient presentation [8].

### 1.7. Treatment

The first therapeutic measure that is required is to stop the administration of amiodarone. The next steps are dependent on the patient’s clinical condition. Some of the studies in the scientific literature recommend observing the patient and revaluating after one month. If the condition is good and the patient is stable, the next evaluation is performed at 3 months, and subsequently at 6 months [8,37,38].

In patients with respiratory failure or significant lung damage, the administration of systemic corticosteroids is indicated [25,37,38]. Prednisolone is used in a dose of 40–60 mg/day. Due to the increased half-life of amiodarone, 2 months of oral corticosteroid treatment is recommended, followed by a dose reduction period, summing up total period of treatment for at least 6 months. Even after administration of this type of treatment, pulmonary recovery, both imaging and functional, is not completely reversible [8,33].

The recommendation to use antifibrotic medication is very recent. Nintedanib is currently indicated for use in pulmonary fibrosis with a progressive phenotype, including in this category pulmonary fibrosis induced by amiodarone [39].

## 2. Case Presentation

The presented cases are from patients admitted to the Pneumology Department of the Mures Clinical County Hospital. Informed consent was obtained from all patients involved in the study. This study was conducted in accordance with the Declaration of Helsinki and approved by the Ethics Committee of Mures Clinical County Hospital, Romania (protocol code (IRB number) 16051, on 7 November 2022).

### 2.1. Case Presentation 1

We present the case of a 67-year-old male patient, with a history of atrial fibrillation, aortic insufficiency III/IV and tricuspid insufficiency I/IV from 2015. The patient was treated with dabigatran etexilate 75 mg, perindopril and amiodarone for one month.

**Figure 1:**
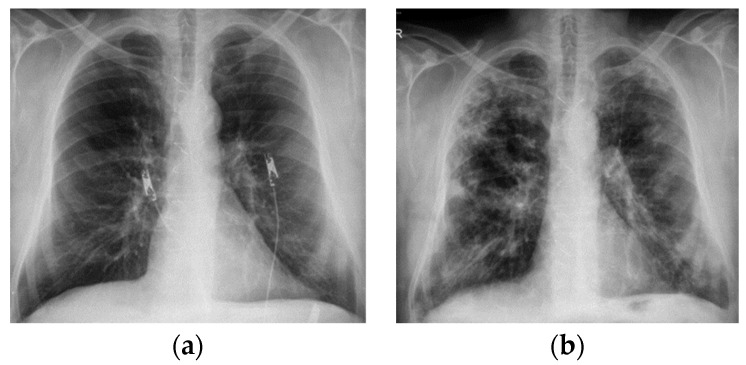
Chest X-ray—2019—Case 1. July 2019: no signs of interstitial damage; October 2019: reticular opacities associated with ground glass opacities with heterogeneous distribution, located subpleural especially. (**a**) July 2019; (**b**) October 2019.

In September 2019, the atrial fibrillation relapsed and was treated with amiodarone, but in October 2019 he came back accusing dyspnea present on rest and clinically presenting hypoxemia. It should be noted that the lung X-ray (Figure 1) from July 2019 is without changes.

**Figure 2:**
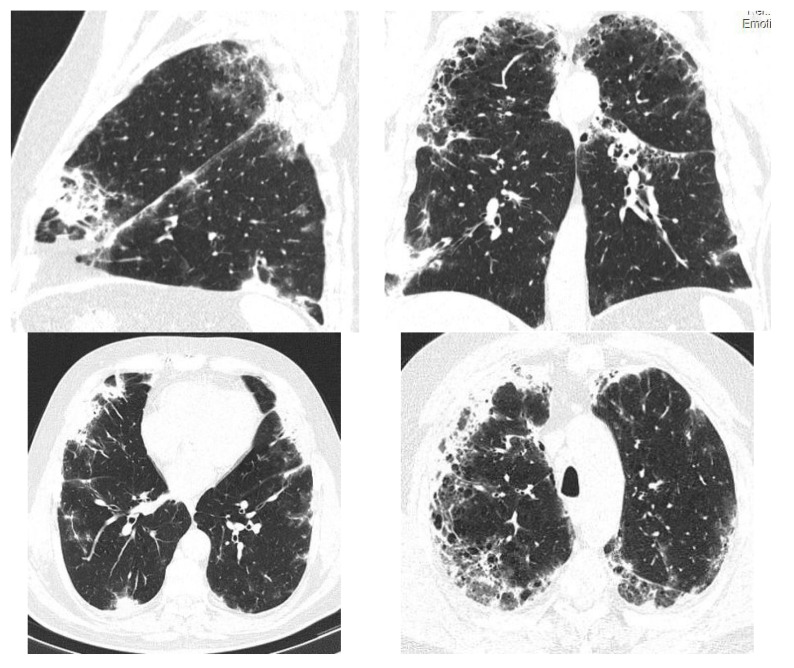

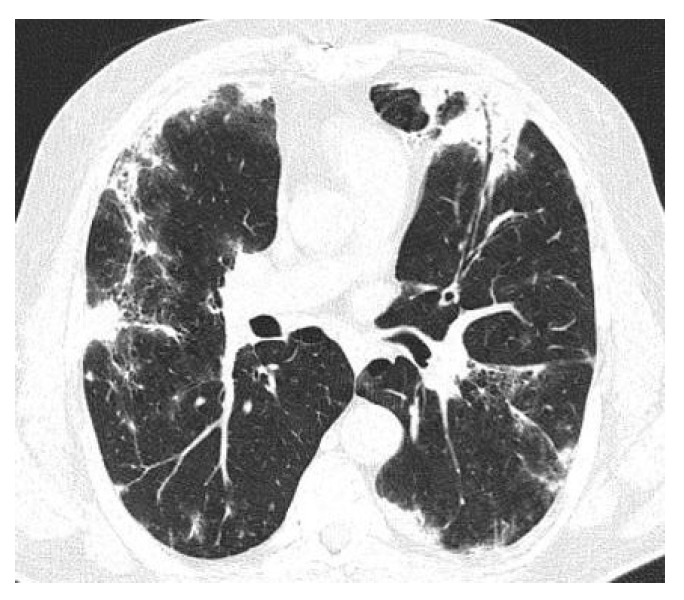
HRCT—July 2019—Case 1. Subpleural honeycombing, nodular lesions, linear fibrosis, traction bronchiectasis, reticulation and ground glass opacities with associated minimal emphysema. Diffuse pulmonary infiltrates in the inferior half.

**Figure 3:**
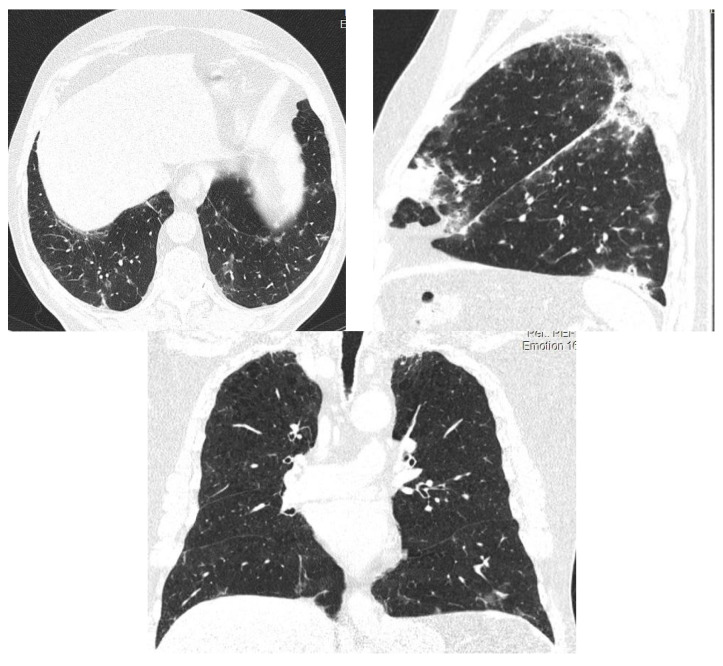
HRCT—2020—Case 1. In comparison to 2019 HRCT, reduced ground glass opacities, minimum bronchiectasis associated with minimum fibrosis and bilateral emphysema.

**Figure 4:**
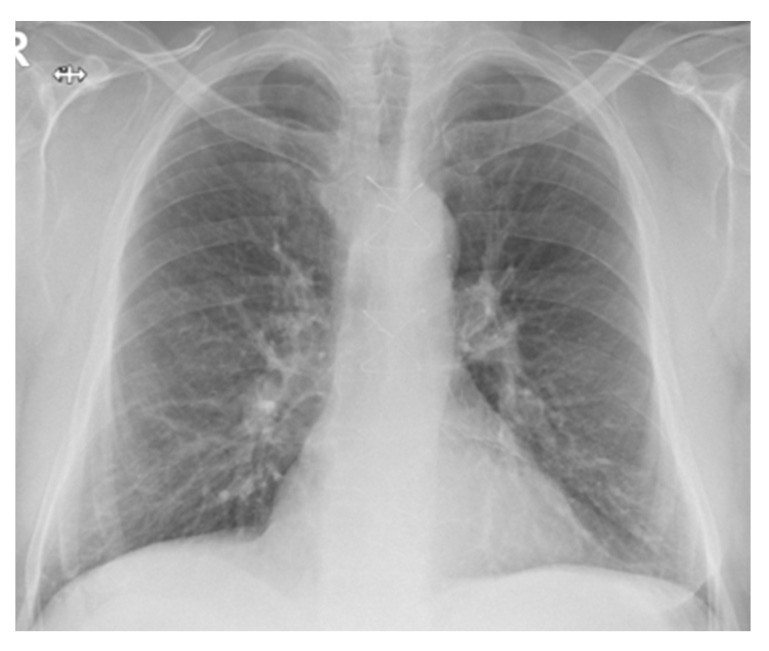
Chest X-ray—2022—Case 1. Diffuse reticular opacities, especially in the basal regions.

The HRCT (Figure 2) showed bilaterally, subpleural honeycombing in the superior half, nodular lesions, linear fibrosis, traction bronchiectasis, reticulation, ground glass opacities, with associated minimal emphysema and bilaterally, subpleural cryptogenic organizing pneumonia foci and diffuse pulmonary infiltrates in the inferior half. The treatment regimen was consequently initiated with corticosteroids (prednisone 45 mg/day and amiodarone discontinuation. As the patient was known to have diabetes, the dose of prednisone was reduced to 20 mg/day for 5 months, and subsequently the treatment with prednisone was gradually stopped. Since the symptoms reappeared, prednisone 15 mg/day continued for another 3 months. Afterwards, the patient had a favorable evolution with 90% oxygen saturation and dyspnea during ordinary activities. In 2020, another high-resolution computed tomography was performed and it revealed a better pulmonary aspect with persistent and reduced ground glass opacities, minimum bronchiectasis associated with minimum fibrosis and bilateral emphysema (Figure 3). The evolution remained favorable until the next evaluation in 2022, without treatment with oral corticosteroids (Figure 4).

### 2.2. Case Presentation 2

We present the case of a 71-year-old male patient, with a history of atrial fibrillation, aortic valve prosthesis and mitral insufficiency from 2016. The patient was treated with amiodarone for 5 years.

The imaging appearance on the thoracic CT was performed 2 years after the initiation of amiodarone treatment, and although the patient was not symptomatic from a respiratory point of view, it describes a more pronounced honeycombing appearance in the right lung field. Functional exploration reveals a restrictive syndrome with FVC 45%, FEV1 71% and an FEV1/FVC ratio of 89. The patient was not referred to a Pulmonology service.

**Figure 5:**
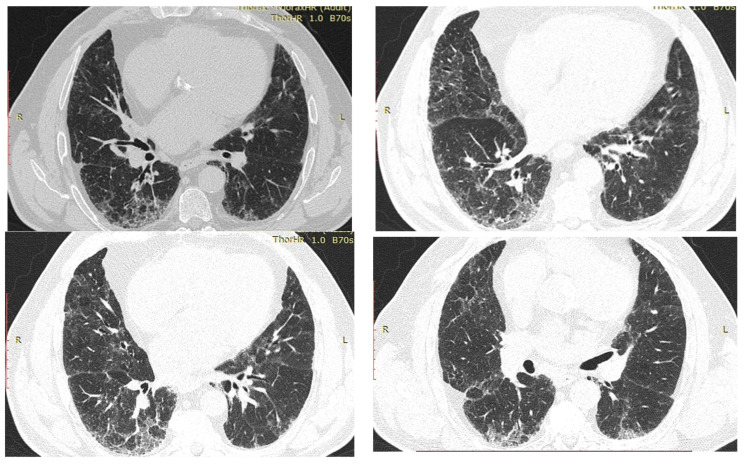
HRCT—2021—Case 2. Reticulation plaques, traction bronchiectasis, subpleural air microcysts. No honeycombing. Probable UIP pattern.

After 5 years of treatment with amiodarone 200 mg/day (2021), the patient was sent for a pulmonary control before performing a coronary angiography procedure. The HRCT examination (Figure 5) reveals an appearance of pulmonary fibrosis with a probable UIP (usual interstitial pneumonia) pattern, with reticulation plaques, traction bronchiectasis, subpleural air microcysts, without the typical appearance of honeycombing, in places with crazy paving.

After this imaging interpretation, at the recommendation of the pulmonologist, the treatment with amiodarone was stopped, and although the patient had no respiratory symptoms, and treatment with metoprolol, acenocumarolum and candesartan was initiated.

Spirometry describes a restrictive syndrome with FVC 64.6%, FEV1 72% and FEV1/FVC of 86.34.

At pulmonary revaluation after 1 year, the patient reported that the 2-month amiodarone treatment had been resumed because the cardiologist intended to perform an ablation procedure. HRCT further describes pulmonary changes with the appearance of probable UIP. Body plethysmography: total resistance 93%, residual volume (RV) 60%, total lung capacity (TLC) 62%, FEV1 78%, DLCO 58. Although the patient presented a progressive fibrotic phenotype, he did not accept to initiate antifibrotic treatment.

### 2.3. Case Presentation 3

We present the case of a 75-year-old female patient, with a history of atrial fibrillation and arterial hypertension. The patient was treated with amiodarone (200 mg/day) for 6 years. The presentation in the Pulmonology Service was made because the patient complained of dyspnea at low/medium efforts.

**Figure 6:**
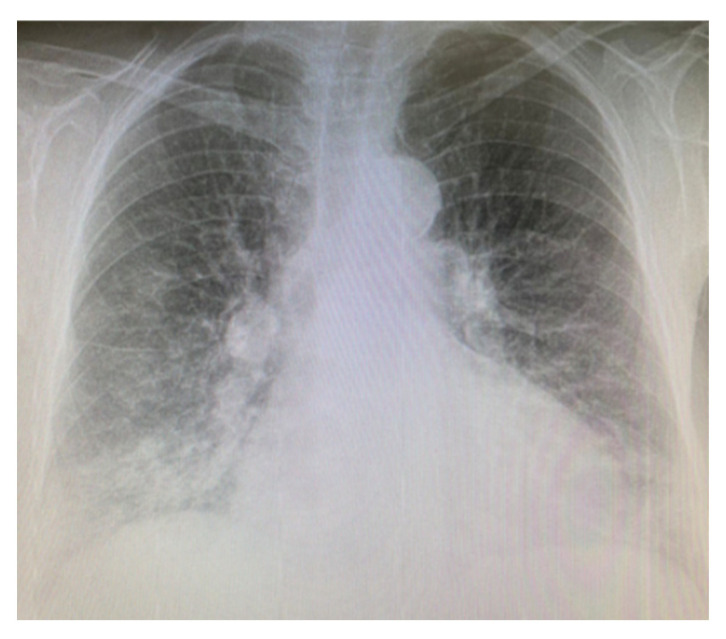
Chest X-ray—2017—Case 3. Nodular and micronodular opacities localized mainly in the lower lobes. Veiling of the pulmonary bases. Reticular opacities on both pulmonary areas. Enlarged cardiac silhouette.

After one year since initiation of amiodarone treatment, a chest X-ray (Figure 6) was initially performed, followed by a native thoracic CT (2017), which revealed in the lower lobes extensive ground-glass areas with bronchiectasis, fibrosis changes in honeycombs, numerous micronodules and centrilobular pulmonary nodules (maximum 5 mm), some with a tree-in bud disposition.

**Figure 7:**
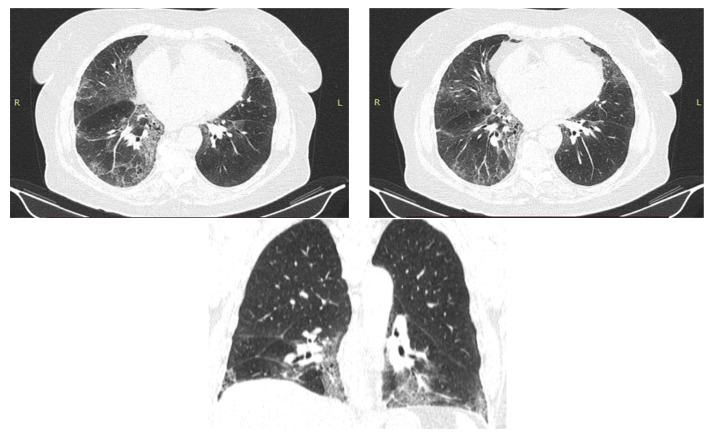
HRCT—2022—Case 3. Extensive areas of pulmonary condensation with ground glass. Bronchiectatic dilations.

The patient did not present then to the Pulmonology Service, and the CT examination (Figure 7) was repeated 5 years later, in 2022. Extensive areas of pulmonary condensation with ground glass appearance predominantly in the basal segments were described bilaterally, associating fibrotic lesions and bronchiectatic dilations.

The pulmonary functional examination revealed normal lung volumes and flows (simple spirometry and body plethysmography), DLCO 80, although the imaging aspect revealed important pulmonary changes of fibrotic type, for which reason it was decided only to monitor the patient and she was directed to cardiology for evaluation the possibility of excluding amiodarone from chronic treatment.

## 3. Discussion and Conclusions

Amiodarone-induced interstitial lung damage includes varying forms of presentation, from mild to moderate/severe [40]. These include organizing pneumonia, interstitial pneumonitis or respiratory failure. The typical presentation of amiodarone-induced lung damage is subacute, with dry cough, progressive dyspnea, low-grade fever and weight loss [25]. Case 2 fits this description, while case 1 presented severe symptomatology with acute respiratory failure, and case 3 presented poor and nonspecific symptomatology. Associated chronic medication or occupational exposure could not be associated with the imaging lesions for any of the patients. The increased risk of developing amiodarone-induced pulmonary fibrosis is directly related to the dose and the duration of the intake. Case 1 followed treatment with 400 mg/day, considered to be a high dose, but for a short period (only one month), and the other two cases followed treatment with a low dose of amiodarone (200 mg/day). Although pulmonary-adverse effects occur at increased cumulative dose associated with long duration of administration, imaging lesions were significant in these patients. Although amiodarone is a potent antiarrhythmic, studies in the specialized literature have demonstrated the occurrence of pulmonary toxicity associated with this treatment [4,21]. The incidence of these complications has decreased considerably with the use of reduced doses, but nevertheless, in some cases, the clinical presentation is acute and may be life-threatening [8,37].

Although the adverse effects produced using amiodarone are known, the adherence of medical staff and patients to the guidelines for monitoring therapy is poor [31,41]. Worldwide, there are medical centers where it is possible to determine the serum level of amiodarone. Values higher than 2.5 mg/L are indicators of a high level of toxicity [31,42]. In our hospital, there is no possibility of this serum’s determination. This determination is useful both for the determination of amiodarone-induced toxicity, in case the patient is symptomatic, or in case of recurrence of an arrhythmia and the possibility of discontinuing the treatment is discussed [31,43]. Differential diagnosis of pulmonary fibrosis induced by amiodarone is made mainly with idiopathic pulmonary fibrosis, left ventricular failure or infectious disease [33,44]. The differential diagnosis in these patients with left ventricular failure is difficult to achieve because cardiac pathology is predominant, and many of the patients have multiple pathological personal antecedents from a cardiological point of view [33,45].

Therefore, preventive and monitoring measures have an important place in the management of these patients. Before starting treatment with amiodarone, patients should be informed of potential adverse effects and prompt reporting of any new respiratory symptoms to their family physician or attending physician [4,9,38].

The assessment carried out at the initiation of amiodarone treatment should include at least a chest X-ray and respiratory function tests, including DLCO [21,45].

None of the patients included in our case series had this assessment performed, which made it difficult to fully assess the extent of lung damage following the period of amiodarone treatment.

Moreover, the assessment at the initiation of amiodarone treatment should also include extrapulmonary evaluation [46]: assessment of lung function, monitoring of liver enzymes or warning patients about the photosensitivity induced by the treatment [29,37]. Because these three patients presented to our Pulmonology Service, we do not have data on extrapulmonary toxicity assessment. The clinical presentation of the three presented cases was different. The first case was presented to pulmonology in an acute manner, with significant lung damage from an imaging point of view and signs of acute respiratory failure. Fortunately, the response to the exclusion of amiodarone from the treatment regimen and the administration of systemic corticosteroids was a very good one. Pulmonary toxicity occurs both in clinically acute forms and in chronic forms [47], such as in cases 2 and 3.

Radiological changes unaccompanied by significant clinical impact can often go unnoticed [1,47]. In cases where imaging damage is accompanied by severe clinical impact, therapeutic treatment with oral corticosteroids is also required. The evolution of case 1 is consistent with the results of the study conducted by Mankikian in 2014, who claimed that, from an imaging point of view, an improvement in alveolar opacities is observed upon HRCT examination in the medium term. This trend is also maintained in the long-term, with an improvement in the severity score on HRCT of 40%, but without restitutio ad integrum [37]. Pulmonary functional exploration highlights a restrictive syndrome. DLCO decreased by 15% advocates pulmonary toxicity in a patient under amiodarone treatment [1,47]. In the first two cases presented, we do not have the DLCO value available, and case 3 presents with a DLCO of 80%, which practically excludes the phenomena of diffusion alteration. Case 2 would have been eligible according to the latest recommendations for antifibrotic therapy, but the patient refused, being scheduled for an ablation procedure.

In cases with no significant clinical impact, the simple exclusion of amiodarone from the treatment scheme is sufficient and no other therapeutic measures are necessary [1,47,48].

Bronchoalveolar lavage was not performed in any of the three cases presented. The diagnosis of pulmonary toxicity induced by amiodarone is difficult, it is a diagnosis of exclusion, based on clinical phenomena of respiratory insufficiency, imaging interstitial-type affection, sometimes even usual interstitial pneumonia, which makes the differential diagnosis with idiopathic pulmonary fibrosis and/or biological [25,46,48]. Early recognition of respiratory complications induced by amiodarone treatment and intensive treatment can cause a favorable evolution of the patient. Any delay in discontinuing amiodarone treatment when there is clinical suspicion may lead to an unfavorable prognosis for the patient [49]. Further imaging and functional monitoring (volumes and respiratory flows) is mandatory.

The prevention of adverse effects is the responsibility of the entire team that interacts with the patient: the attending physician, the one who prescribes the treatment, primary care physician, specialist physician and pharmacist [50]. The multidisciplinary approach remains essential to improving the quality of life and the patient’s outcome. Effective follow-up of the patient after initiation of amiodarone therapy involves responsibility on the part of the entire medical team as well as the patient [51]. Current information and effective communication between patient and doctor are essential for further development [31].

## Figures and Tables

**Table 1:** Main differential diagnostics of amiodarone-induced pulmonary fibrosis.

Idiopathic pulmonary fibrosis	Usual interstitial pneumonia (UIP). High-resolution CT often shows honeycomb changes, traction bronchiectasis and a reticular pattern that is predominantly in the periphery of the lower lobes.
Hypersensitivity pneumonitis	Exposure history. Specific IgG antibodies. Pulmonary infiltrates and suspected nonspecific interstitial pneumonia or idiopathic pulmonary fibrosis.
Infectious pathologies	Purulent sputum, unilateral localization. Procalcitonin value elevated.
Heart failure. Acute cardiogenic pulmonary oedema	Kerley lines and peri bronchial oedema. Subpleural patchy areas in pulmonary oedema.
BOOP	Procalcitonin value unchanged.

## Data Availability

Not applicable.
